# The mitochondrial genome of the land snail *Camaenella platyodon* (Pfeiffer, 1846) (Stylommatophora, Camaenidae)

**DOI:** 10.1080/23802359.2019.1644559

**Published:** 2019-07-25

**Authors:** Mei-Ling Hu, Pei Wang, Yu Chen, Ming-Zhe Zhang, Ping-Shan Yang, Jun-Hong Lin, Wei-Chuan Zhou

**Affiliations:** a Key Laboratory of Molluscan Quarantine and Identification of GACC, Fuzhou Customs, Fujian, China;; b Ningde Customs, Fujian, China;; c Hangzhou Customs, Zhejiang, China;; d College of Plant Protection, Fujian Agriculture and Forestry University, Fuzhou, China

**Keywords:** *Camaenella platyodon*, Camaenidae, mitochondrial genome, phylogeny

## Abstract

The complete mitochondrial genome (mitogenome) of Chinese endemic snail *Camaenella platyodon* (Pfeiffer, 1846) has been sequenced and annotated in this study. The entire circular genome is 13,985 bp in size and represents the third camaenid mt genome, with 2 ribosomal RNA genes, 22 transfer RNA genes, 13 protein-coding genes. All of genes are divided into two groups, including 24 genes on the majority coding strand (J strand) and others on the minority coding strand (N strand). Phylogenetic analysis of 13 protein-coding genes suggests that *C*. *platyodon* is closely related to the species in family Camaenidae.

The land snail *Camaenella platyodon* (Pfeiffer, 1846) can destroy crops seriously, spread zoonotic food borne parasitic disease, and cause substantial damage to human and animal health (Butcher and Grove 2005; Qian and Zhou 2014; Zhou et al. 2007). So far, there is not any report on mitogenome of *C. platyodon*. Here, we sequenced the complete mitogenome of this snail and assessed phylogenetic position in order to offer more worthwhile information for phylogeny and methods in molecular identification.

Adult snail was collected from Hainan Bayi Farm, China (19°27′11.39″N, 109°17′38.43″E) and stored at −20 °C for long term at the Herbarium of plant pests, Fuzhou customs, Fujian, China (Number: FJIQBC20431). Total genomic DNA was extracted from the pedal muscle tissue of single individual using the DNeasy Blood and Tissue kit (Qiagen) according to the manufacturer’s instructions. The whole mitogenome was sequenced on the Illumina Hiseq 2500 platform at Berry Genomics, Beijing. The tRNA genes were identified with tRNAscan-SE Search Server v.1.21 (Lowe and Eddy 1997) and DOGMA (Wyman et al. 2004). The PCGs and rRNA genes were annotated using BLAST in Genbank with published available mitochondrial sequences of terrestrial snails (Wang et al. 2014; Yang et al. 2014; Deng et al. 2016; He et al. 2016). Phylogenetic analyses were performed using maximum-likelihood (ML) method.

The entire circular genome was 13,985 bp long (GenBank: MH362759), containing two ribosomal RNA genes, 22 transfer RNA genes, 13 protein coding genes. 24 genes were encoded on the majority coding strand (J strand) except other 13 genes (*tRNA^Gln^*, *tRNA^Leu(UUR)^*, *tRNA^Asn^, tRNA^Arg^*, *tRNA^Glu,^ tRNA^Met^, tRNA^Ser(UCN)^*, *tRNA^Thr^*, *ATP6*, *ATP8*, *ND3*, *COIII*, and *SrRNA*) oriented on the minority coding strand (N strand). The nucleotide composition of the whole genome was biased toward adenine and thymine, accounting for71.97%. Gene overlaps with a total of 329 bp had been found at 19 gene junctions, and the longest overlap (59 bp) existed between *ND5* and *ND1*. In addition, there were 132 nucleotides dispersed in nine intergenic spacers, the largest of which was 43 bp between *COIII* and *tRNA^Ile^*. All PCGs started strictly with ATN (three with ATG, five with ATT, and five with ATA). Conventional stop codons TAA and TAG had been assigned to all of PCGs except ND2 with T—. The length of tRNA genes ranged from 55 to 66 bp. The length of *lrRNA* and *srRNA* were determined to be 999 bp and 699 bp, respectively. The absence of control region was consistent with other snails from Gastropoda (Wang et al. 2014; Yang et al. 2014; Deng et al. 2016).

The ML tree (Figure 1) presented 17 major clades containing the Bradybaenidae, Camaenidae, Hygromiidae, Helicidae, Polygyroidae, Urocoptoidae, Cerionidae, Orthalicidae, Succineidae, Clausiliidae, Achatinellidae, Pupillidae, Vertiginidae, Achatinidae, Linacoidae, Lymnaeidae, and Aplysiidae. The five bradybaenid species and three camaenid species are close to each other and the sister-group relationship was also recovered. Three species in the Camaenidae formed a monophyletic group. However, the phylogeny of Camaenidae, Helicidae, and Bradybaenidae are complicated and have not been fully resolved; systematic and phylogenetic studies based on analyses of morphological and molecular markers have produced inconsistent results (Scott 1996; Hirano et al. 2014). More information of related species needs to be prepared to assess the phylogenetic relationship of these three families.

**Figure 1.**
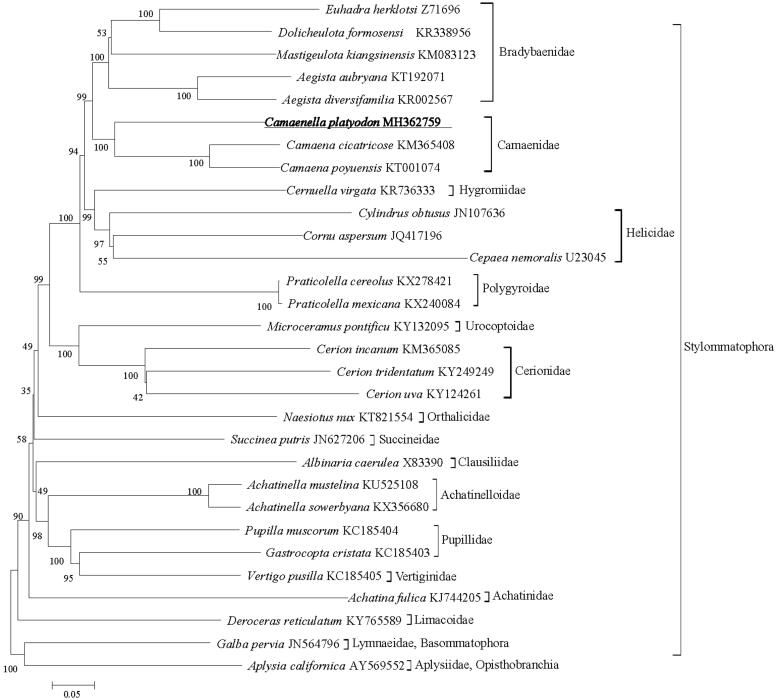
Phylogenetic tree inferred using ML method based on 13 protein-genes. The tree is rooted with *Aplysis californica* and *Galba pervia*. Numbers on or under the nodes represent bootstrap values.
